# Tags Attached to the Centrosomal Protein SPD-5 Can Disrupt Centrosome Flattening During Anaphase of the 1-cell *C. elegans* Embryo

**DOI:** 10.17912/micropub.biology.002285

**Published:** 2026-09-23

**Authors:** Robert S. Mirabello, Mohammad M. Rahman, Abigail Ruddick, Danielle R. Hamill, Bruce Bowerman, Richa Maheshwari, Orna Cohen-Fix

**Affiliations:** 1 The Laboratory of Biochemistry and Genetics, National Institute of Diabetes and Digestive and Kidney Diseases, Bethesda, MD, United States; 2 Department of Biology, University of Oregon, Eugene, OR, United States; 3 Department of Biological Sciences, Ohio Wesleyan University, Delaware, OH, United States

## Abstract

Centrosomes promote mitotic spindle assembly by nucleating microtubules from the peri-centriolar material (PCM). At mitotic exit, centrosomes disassemble and the PCM disperses. In the 1-cell *C. elegans* embryo, polarity cues result in distinct disassembly patterns of the anterior vs. posterior centrosome. Here, we show that commonly used alleles of GFP-tagged SPD-5 , a major PCM protein, can interfere with posterior centrosome flattening during disassembly. We also report the development of endogenous super-folder GFP:: SPD-5 (sfGFP:: SPD-5 ) that exhibits normal centrosome flattening. This highlights the ability of tags to interfere with cellular structures and processes, and provides a new tool to symmetrize centrosome disassembly.

**Figure 1. Tagging SPD-5 with fluorescent proteins can prevent posterior centrosome flattening in the 1-cell C. elegans embryo:** (A) i-viii: Confocal fluorescent microscopy images of the indicated fluorescently tagged SPD-5 in 1-cell *C. elegans* embryos at metaphase and at 4 and 5 minutes after metaphase. The alleles indicated in white are untagged. Embryos are all oriented with anterior end facing left. The outline of the embryo in panel i is shown for orientation purposes. The strains in each panel are: i: JLF359, ii: OCF176, iii: OCF259, iv: OCF187, v: OCF218, vi and vii: OCF221, viii: OCF258. ix: A representative example of a time course of a posterior centrosome that exhibits flattening (JLF359; RFP ^end^ ) from metaphase to the end of centrosome disassembly. Absolute fluorescence intensity range is the same across all panels. Scale bar: 3 µm. x: As in ix, but for a posterior centrosome that does not exhibit flattening (OCF176; GFP1 ^end^ ). (B) Indirect immunofluorescence of SPD-5 using anti- SPD-5 antibodies (in green) of three embryos in anaphase to telophase (from top to bottom), as described in Hamill et al (2002). DNA is stained by propidium iodide (red). Scale bar: 10 µm (C) Length of posterior PCM at 4 minutes after metaphase, for strains with endogenous and/or transgenic tagged SPD-5 , as indicated. N/A indicates that the strain did not contain a transgene. Centrosomes where the PCM was at least 5 µm long were considered flattened. n= number of embryos analyzed. **p=0.0015 for RFP ^end^ vs GFP1 ^end^ ; ***p=0.0003 for RFP ^end^ vs GFP2 ^end^ ; ns: p≥0.05. Statistical analysis was done using one-way ANOVA Kruskal-Wallis test with Dunn's multiple comparisons correction. Error bars: mean ± SD. (D) Linker sequences between the FP C-terminus and SPD-5 's N terminus, the linker's protein motifs (if present), and method of insertion into the genome for each of the *spd-5* alleles shown in panel A. (E) Area of posterior centrosomes at metaphase, brood size and embryonic viability for the strains described in panel A plus a control strain, OCF15 (last row), in which endogenous *spd-5* is not tagged and there is no transgene. For metaphase centrosome area, n= 9-11. There were no statistically significant differences between any of the strains and the RFP strain as determined by one-way ANOVA Brown-Forsythe test with Dunnett's multiple comparisons correction (the control strain is untagged and thus could not be measured). Values represent mean ± SD. For brood size, n= 9-21 individual L4s. None of the strains were statistically different from the control, except the strain that only expressed a GFP:: SPD-5 transgene (GFP3 ^trans^ ; OCF187), which showed a slightly higher brood size (p= 0.0116) as determined by one-way ANOVA Kruskal-Wallis test with Dunn's multiple comparisons correction. Values represent mean ± SD. Embryonic viability was determined in triplicates. None of the strains were statistically different from the control. Values represent mean ± SD.

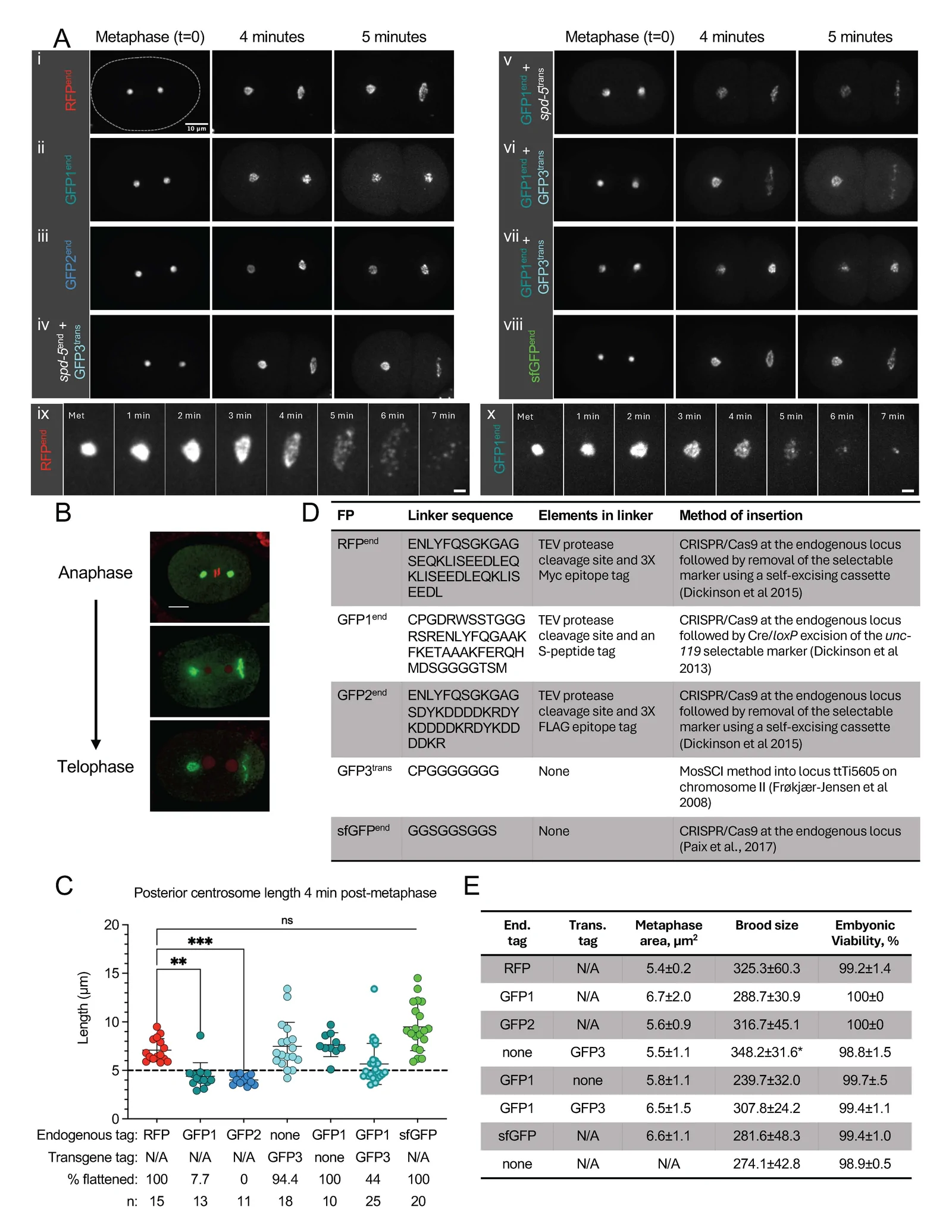



## Description

Centrosomes, composed of barrel-like centrioles surrounded by peri-centriolar material (PCM), are dynamic structures that help build mitotic spindles in metazoan cells by promoting microtubule nucleation (Nigg and Raff, 2009). Centrosomes increase in size until metaphase as the PCM expands, a process known as centrosome maturation. In *C. elegans* , SPD-5 is a coiled-coil protein that serves as a major PCM protein by acting as a scaffold (Hamill et al., 2002). SPD-5 's phosphorylation by the kinase PLK-1 drives SPD-5 multimerization and recruitment of downstream clients, resulting in centrosome maturation (Wueseke et al., 2016; Woodruff et al., 2017; Cabral et al., 2019; Ohta et al., 2021; Nakajo et al., 2022; Rios et al., 2024; Ohta et al., 2026). At the end of mitosis, the PCM is dismantled in a process known as centrosome disassembly by the combined action of phosphatases and microtubule-mediated forces (Woodruff et al., 2014; Conduit et al., 2015; Enos et al., 2018). In *C. elegans* , dephosphorylation weakens SPD-5 's intermolecular interactions, allowing microtubule-mediated forces to rip apart the PCM (Enos et al., 2018; Magescas et al., 2019; Mittasch et al., 2020). In the 1-cell embryo, centrosome disassembly is asymmetric; in telophase, the anterior centrosome remains spherical while the posterior centrosome flattens due to microtubule-pulling forces (Strome and Wood, 1983; Keating and White, 1998; Hamill et al., 2002; Severson and Bowerman, 2003). Similar flattening is also observed in other organisms, such as the nematode *Ascaris megalocephala* (Boveri, 1900), the surf clam *Spisula solidissima* (Dan and Ito, 1984) and across multiple orders of sea urchins (Boveri, 1900; Dan, 1979; Endo, 1980; Paweletz et al., 1984). The role of centrosome flattening is not known.

In the *C. elegans* embryo, the centrosome is surrounded by a membrane reticulum called the centriculum (Maheshwari et al., 2023; Maheshwari et al., 2026). To follow the fate of the centriculum after metaphase, we visualized centrosomes by live fluorescent confocal microscopy using SPD-5 tagged with a fluorescence protein (FP). SPD-5 is commonly used for this purpose, and we had several such constructs on hand that have been previously used in the field [e.g. (Cabral et al., 2019; Magescas et al., 2019; Garbrecht et al., 2021; Magescas et al., 2021; Holzer et al., 2022; Garcia-Baucells et al., 2025)]. Using endogenously expressed tagRFP-T:: SPD-5 (allele: *wow36* (Magescas et al., 2019), henceforth “RFP ^end^ ”), we observed the expected flattening of the posterior but not anterior centrosome ( Fig. 1A i and ix), similar to the centrosome flattening observed by indirect immunofluorescence [ Fig. 1B and (Hamill et al., 2002; Severson and Bowerman, 2003; Enos et al., 2018)], and consistent with this allele's behavior in previous publications (Magescas et al., 2019; Rios et al., 2024; Schreiner et al., 2025). Unexpectedly, in strains expressing two different endogenously tagged GFP:: SPD-5 constructs ( *vie26* (Cabral et al., 2019) and *wow52* (Magescas et al., 2019), referred to here as GFP1 ^end^ and GFP2 ^end^ , respectively), the flattening of the posterior centrosome was abolished (Figs. 1Aii, iii and x). Despite the flattening defect, centrosomes in the GFP1 ^end^ and GFP2 ^end^ strains disassembled as in the RFP strain, as determined by the complete dispersal of SPD-5 fluorescence surrounding the centrioles (Figs 1Aix and x). To quantify the defect in flattening, embryos were imaged every minute starting at metaphase. Centrosome flattening began 4 minutes after metaphase and increased through 5 and 6 minutes. At 5 minutes, the PCM had begun disassembling into puncta that were occasionally too faint or disperse to measure. We thus quantified posterior centrosome flattening by measuring the length of the PCM long axis at 4 minutes after metaphase ( Fig. 1C ).

We classified any centrosome with a ≥ 5.0 µm PCM at 4 minutes post-metaphase as being flattened. In a strain expressing RFP ^end^ , posterior centrosomes flattened in 100% of embryos imaged (Figs. 1Ai, ix and 1C). In contrast, posterior centrosomes failed to flatten in the two GFP:: SPD-5 strains, GFP1 ^end^ and GFP2 ^end^ , and posterior PCM lengths in these strains were significantly shorter than posterior RFP ^end^ centrosomes (Figs. 1Aii, iii, x, and 1C). In addition to the fluorescent tag, RFP ^end^ , GFP1 ^end^ and GFP2 ^end^ also differ in the linkers between the FP and SPD-5 ( Fig. 1D ). To determine whether these three constructs lead to a general difference in PCM morphology, we measured centrosome areas at metaphase (when centrosomes are spherical) and found no significant differences between the three strains ( Fig. 1E ).

We also examined centrosome flattening in a strain expressing GFP:: SPD-5 as a transgene, ( *ltSi1141* (Woodruff et al., 2015), termed GFP3 ^trans^ ), in the presence of untagged endogenous SPD-5 . GFP3 ^trans^ 's linker sequence is much shorter than those in GFP1 ^end^ and GFP2 ^end^ ( Fig. 1D ) but the GFP itself is identical between all three strains. Interestingly, nearly all centrosomes in GFP3 ^trans^ flattened ( Fig. 1C ). This is consistent with Enos et al., (2018) and Erpf et al., (2019) who observed posterior centrosome flattening in strains expressing the same construct. Given the presence of the untagged endogenous SPD-5 in GFP3 ^trans^ , we hypothesized that either the untagged endogenous protein was rescuing flattening by diluting GFP3 ^trans^ , or GFP3 ^trans^ itself permitted centrosome flattening.

To test if untagged SPD-5 can rescue centrosome flattening in the presence of GFP:: SPD-5 , we combined GFP1 ^end^ with untagged transgenic SPD-5 . 100% of centrosomes flattened in this strain (Figs. 1Av and 1C), suggesting that untagged SPD-5 can rescue centrosome flattening that is inhibited by the GFP:: SPD-5 constructs used here. We next examined whether GFP3 ^trans^ itself is permissive to centrosome flattening by combining it with GFP1 ^end^ . We reasoned that if GFP3 ^trans^ permits flattening in the presence of GFP1 ^end^ , then GFP3 ^trans^ behaves as an untagged SPD-5 . Interestingly, in this GFP1 ^end^ + GFP3 ^trans^ strain, 44% of centrosomes flattened (Figs. 1Avi and vii, and 1C). GFP and other FPs can dimerize and perturb organelle morphology (Zacharias et al., 2002; Snapp et al., 2003; Costantini et al., 2012). In our case, the expression of GFP1 ^end^ and GFP2 ^end^ as the sole source of SPD-5 may have increased inter-GFP:: SPD-5 interactions, thus preventing anaphase SPD-5 dispersal and consequently, centrosome flattening. The presence of untagged SPD-5 would increase the average distance between GFP1 ^end^ or GFP2 ^end^ molecules, reducing their ability to interact. GFP3 ^trans^ has a much shorter linker than GFP1 ^end^ or GFP2 ^end^ ( Fig. 1D ); the short linker might constrain GFP3 ^trans^ 's dimerization, thus perturbing centrosome flattening to a lesser degree than GFP1 ^end^ or GFP2 ^end^ . Furthermore, in strains expressing both GFP3 ^trans^ and GFP1 ^end^ , posterior centrosome flattening might occur in embryos with higher relative levels of GFP3 ^trans^ expression, while in embryos with higher relative GFP1 ^end^ expression, flattening might be inhibited.

Thus far, the only strains that exhibited posterior centrosome flattening were RFP ^end^ and those expressing SPD-5 transgenes. We therefore sought to construct a strain expressing endogenously tagged “green” SPD-5 that exhibited wild type centrosome flattening without a transgene. To this end, we used super-folder GFP (sfGFP), which has been shown to be more monomeric than GFP by gel filtration, crystallography, and cell-based assays (Zacharias et al., 2002; Pedelacq et al., 2006; Costantini et al., 2012). Of note, recent reports using cell-based assays reported that sfGFP might dimerize, although less so than many other FPs, like tagRFP-T (Cranfill et al., 2016; Stoddard and Rolland, 2019; Fraikin et al., 2025). Using CRISPR-Cas9 gene editing, we tagged SPD-5 endogenously, affixing sfGFP to SPD-5 's N-terminus via a 9 amino acid linker reminiscent of GFP3 ^trans^ 's linker ( Fig. 1D ). In this strain, where sfGFP:: SPD-5 is the only source of SPD-5 , 100% of posterior centrosomes flattened after metaphase (Figs. 1Aviii and 1C). Thus, we successfully constructed an endogenous green FP-tagged SPD-5 that retains normal centrosome morphology during late mitosis.

Whether FP dimerization is what led to a defect in centrosome flattening in the strains expressing GFP1 ^end^ and GFP2 ^end^ remains an open question. Beyond dimerization, FPs could disrupt PCM disassembly by interfering with microtubule nucleation or SPD-5 's accessibility to phosphatases, as both microtubules and phosphatases are required for PCM disassembly (Enos et al., 2018; Magescas et al., 2019; Mittasch et al., 2020). The nature of the linker ( Fig. 1D ) could have also affected the properties of the FP:: SPD-5 fusion proteins. It also remains unknown whether inhibiting centrosome flattening leads to a developmental defect; neither brood size nor embryonic viability was significantly different in strains without posterior centrosome flattening compared to a control expressing untagged SPD-5 ( Fig. 1E ). Either flattening, or lack thereof, has no functional importance, or the embryo has robust mechanisms to compensate for this defect. Thus, the consequences of abolishing posterior centrosome flattening should be examined further. In this regard, if the GFP1 ^end^ and GFP2 ^end^ SPD-5 fusion proteins do affect centrosome-related processes, there is a concern that they may exhibit synthetic genetic interactions under conditions that partially compromise centrosome function, even if these conditions, on their own, do not exhibit a centrosome defect. Finally, the GFP1 ^end^ and GFP2 ^end^ alleles provide useful tools that inhibit centrosome flattening without global disruption of microtubules, phosphatases, or motor proteins. Our study further underscores that PCM disassembly is sensitive to the properties of its constituent proteins.

## Methods

***C. elegans* Strains**

The *C. elegans* strains used in this study were derived from the N2 strain (Bristol; Brenner, 1974) and its derivatives and are listed under Reagents. Strain OCF259 was created by crossing OCF15 with strain JLF361, described previously (Magescas et al., 2019). Strains were maintained at 20°C on *E. coli* OP50 lawns seeded on MYOB agarose using standard methods (Brenner, 1974).

**CRISPR-Cas9**

Tagging of *spd-5* with *sfGFP* was mostly done according to previously published methods (Paix et al., 2017). The *sfGFP* sequence was inserted into a *pUC-GW-Amp* vector by Azenta Life Sciences. The sequence is as follows, with linkers in bold and introns in lowercase:

**GGTGGTTCCGGTGGTTCTGGTGGTTCT** GTCAGCAAAGGAGAAGAACTTTTCACTGGAGTTGTCCCAATTCTTGTTGAATTAGATGGTGATGTTAATGGGCACAAATTTTCTGTCCGTGGAGAGGGTGAAGGTGATGCTACAAACGGAAAACTCACCCTTAAATTTATTTGCACTACTGGAAAACTACCTGTTCCATGGgtaagtttaaacatatatatactaactaaccctgattatttaaattttcagCCAACACTTGTCACTACTCTGACCTATGGTGTTCAATGCTTTTCCCGTTATCCGGATCACATGAAACGGCATGACTTTTTCAAGAGTGCCATGCCCGAAGGTTATGTACAGGAACGCACTATATCTTTCAAAGATGACGGGACCTACAAGACGCgtaagtttaaacagttcggtactaactaaccatacatatttaaattttcagGTGCTGAAGTCAAGTTTGAAGGTGATACCCTTGTTAATCGTATCGAGTTAAAAGGTATTGATTTTAAAGAAGATGGAAACATTCTCGGACACAAACTCGAGTACAACTTTAACTCACACAATGTATACATCACGGCAGACAAACAAAAGAATGGAATCAAAGCTgtaagtttaaacatgattttactaactaactaatctaatttaaattttcagAACTTCAAAATTCGCCACAACGTTGAAGATGGTTCCGTTCAACTAGCAGACCATTATCAACAAAATACTCCAATTGGCGATGGCCCTGTCCTTTTACCAGACAACCATTACCTGTCGACACAATCTGTCCTTTCGAAAGATCCCAACGAAAAGCGTGACCACATGGTCCTTCTTGAGTTTGTAACTGCTGCTGGGATTACACATGGCATGGATGAGCTCTACAAA **GGTGGTTCCGGTGGTTCTGGTGGTTCT**

Because ssDNA repair templates have been shown to be more efficient than dsDNA, we used repair template primers with phosphorothioate linkages introduced for the first 5 nucleotides on the 5' left homology arm, permitting digestion with T7 exonuclease to create a single-stranded repair template (Noteborn et al., 2020). The sequences of the primers were as follows:

Forward primer, with 120 bp *spd-5* promoter (up to ATG) homology arm and 20 bp homology with *sfGFP* N-terminus, in bold. Asterisks indicate phosphorothioate bond:

T*G*C*T*G*AAGCTTCAAATTTTGCAACCTCCTGTTCATTTTGACTCAAAACTCTTAATCCCAAAAAAACGCTCAATTTTGTTTTCGAACCCGTTTCTTGTTTCAGAAAACTTCGCGTTAAATG **GTCAGCAAAGGAGAAGAACT**

Reverse primer, with 145 bp *spd-5* coding sequence homology arm and 20 bp homology with *sfGFP* C-terminal linker, in bold:

CTGACAATACTTGCTGTTGAGTGGCACTGGTCGAAGACGTTCTTTTGTCTCCTTCAACATTCAGAACTGGTTGCGACATAGATCTTCGTGGCTGGCCCTCGACATGCTCGAGATTGGAGTCTTCATTAAGAACTGAGTTGTCTTC **AGAACCACCAGAACCACCGG**

Our gRNA sequence to target the *spd-5* start codon was: GGATAATTCTGTGCTCAACG

**Microscopy**

Gravid adults were immobilized on a cover slip with 50 mM levamisole in standard M9 buffer and dissected with hypodermic needles to release their embryos. The cover slip was then transferred to a pad of 2% agarose in standard M9 buffer on a glass slide. Images were taken using a Nikon confocal Ti2 microscope with a Yokogawa CSU-X1 spinning disk and a Photometrics Prime 95B camera using a Nikon oil 60×1.40 NA Apo Plan objective. Images were captured using Nikon Elements software version 5.21.03. Centrosomes were imaged across a 20-µm slice at z=1 µm intervals (21 total images per stack) and at 1-minute intervals starting around metaphase until the approximate completion of centrosome disassembly. Indirect immunofluorescence was as described in Hamill et al, 2002.

**Image Analysis**

All images were analyzed using Fiji (Schindelin et al., 2012; https://imagej.net/ij/ ).

**Measurements**

To measure PCM length, a maximal projection was created using all 21 slices of each image. The timepoint at which metaphase occurs was determined by centrosome shape and positioning: mitotic spindle length (i.e. distance between centrosomes) is around 15 microns at metaphase (Greenan et al., 2010). Metaphase centrosomes are also circular and static; once anaphase begins, the PCM deforms and the centrosomes begin regression to the embryonic poles. At four minutes post-metaphase, the boundary of the posterior centrosome was traced by hand using Fiji's freehand selection tool. The mean and minimum values inside this hand-traced object were recorded, and the average of these two values was set as a minimum threshold to highlight the pixels of the centrosome. The length of the PCM was measured as the length of its longest axis.

For centrosome area, the same tracing and thresholding protocol was followed as PCM length. Area was determined rather than length (namely diameter) to avoid ambiguities in the placement of the line to measure diameter. Once thresholding had determined the pixels constituting the centrosome, this area was measured.

For brood size, single L4s were placed on OP50 *E. coli* lawns seeded on MYOB agarose in a small petri dish (35x10mm). OP50 *E. coli* lawns were ensured to be in the center of the petri dish to prevent worms from straying close to the outer walls. Each worm's progeny was counted by removing its hatched larvae from the dish until no new progeny were laid. Parents were often sequentially transferred to 1 or 2 plates to prevent plates from getting too crowded with progeny.

For embryonic viability, three MYOB plates with OP50 were inoculated with 10 gravid adults each (collected the day before as L4 larvae) and incubated at 20°C. Worms were allowed to lay eggs for 2 hours after which they were removed. The percent of eggs that hatched was first determined after 30 additional hours at 20°C, and the plates were inspected again 24 hours later.

**Statistical Analyses**

All analyses were done using GraphPad Prism [Version 10.6.1 (799)]. D'Agostino-Pearson, Anderson-Darling, Shapiro-Wilk, and Kolmogorov-Smirnov tests were used to test for normality of distributions. Standard deviations (SDs) for all datasets were measured. When samples were normal and had variable SDs, Brown-Forsythe and Welch's ANOVA tests, with Dunnett's T3 multiple comparisons test with individual variances computed for each comparison, were used. When datasets were not normal (nonparametric), the Kruskal-Wallis test with Dunn's correction for multiple comparisons was used.

## Reagents

Reagents

**Table:** 

**Strain name**	**Referred to in this study**	**Genotype**	**Reference**
JLF359	RFP ^end^	*spd-5 (wow36[tagRFP-T::3xmyc:: spd-5 ] I *	Magescas et al., 2019
OCF15 ^*^	N/A	*unc-119 ( ed3 ) III; ocfIs2 [ pie-1 p::mCherry::SP12:: pie-1 3'UTR + unc-119 (+)] *	Joseph-Strauss et al., 2012
OCF176 ^*^	GFP1 ^end^	*spd-5 ( vie26 [GFP:: spd-5 ::loxP]) I;* *his-72 ( erb77 [ his-72 ::linker::mTurquoise2]) III; unc-119 ( ed3 ) III; ocfIs2 [ pie-1 p::mCherry::SP12:: pie-1 3'UTR + unc-119 (+)] *	Maheshwari et al., 2023
OCF187	untagged *spd-5* ^end^ + GFP3 ^trans^	* ltSi1141[(pOD1021/pVV103) spd-2p::GFP:: spd-5 (re-encoded) + Cbr-unc-119 (+)] II; unc-119 ( ed3 ) III; ocfIs2 [ pie-1 p::mCherry::SP12:: pie-1 3'UTR + unc-119 (+)] *	Maheshwari et al., 2026
OCF218	GFP1 ^end^ + untagged *spd-5* ^trans^	*spd-5 ( vie26 [GFP:: spd-5 ::loxP]) I; ltSi1129[(pZZ2) spd-2p:: spd-5 (re-encoded) + Cbr-unc-119 (+)] II; unc-119 ( ed3 ) III; ocfIs2 [ pie-1 p::mCherry::SP12:: pie-1 3'UTR + unc-119 (+)] *	Maheshwari et al., 2026
OCF221	GFP1 ^end^ + GFP3 ^trans^	* ltSi1141[(pOD1021/pVV103) spd-2p::GFP:: spd-5 (re-encoded) + Cbr-unc-119 (+)] II; spd-5 ( vie26 [GFP:: spd-5 ::loxP]) I; unc-119 ( ed3 ) III; ocfIs2 [ pie-1 p::mCherry::SP12:: pie-1 3'UTR + unc-119 (+)] *	Maheshwari et al., 2026
OCF258	sfGFP ^end^	*spd-5 (ocf110[sfGFP:: spd-5 ]) I; unc-119 ( ed3 ) III; ocfIs2 [ pie-1 p::mCherry::SP12:: pie-1 3'UTR + unc-119 (+)] *	This study
OCF259	GFP2 ^end^	*spd-5 (wow52[GFP::3xflag:: spd-5 ]) I; unc-119 ( ed3 ) III; ocfIs2 [ pie-1 p::mCherry::SP12:: pie-1 3'UTR + unc-119 (+)] *	This study

^*^ Available from the Caenorhabditis Genetics Center

**Notes on strains**

^1^ The tagRFP-T includes short insertions (S2_S2delinsVSKGE/H230_K231insKLNGMDELY) from EGFP's termini, as determined by sequencing. This is the predominant variant of tagRFP-T, according to FPbase (https://www.fpbase.org/protein/tagrfp-t/).

^2^ The version of GFP present in all strains in this paper, as determined by sequencing, is original GFP from *Aequorea victoria* with two mutations: S65C—a common mutation in *C. elegans* GFP for improved photostability—and Q80R—a common “neutral” mutation (Tsien, 1998; Green et al., 2008).

^3^ The GFP:: SPD-5 allele in GFP3 ^trans^ , *ltSi1141* , is frequently referred to in the literature as *ltSi202* , including in Woodruff et al., 2015. This allele originated in the Oegema lab, which reports the correct allele for this construct is *ltSi1141* (KO, personal communication).
